# A Dynamic Combinatorial Approach for Identifying Side Groups that Stabilize DNA-Templated Supramolecular Self-Assemblies

**DOI:** 10.3390/ijms16023609

**Published:** 2015-02-06

**Authors:** Delphine Paolantoni, Sonia Cantel, Pascal Dumy, Sébastien Ulrich

**Affiliations:** Institut des Biomolécules Max Mousseron (IBMM), UMR 5247 CNRS-Université de Montpellier, ENSCM, Ecole Nationale Supérieure de Chimie de Montpellier, 8 Rue de l’Ecole Normale, Montpellier Cedex 5 34296, France; E-Mails: delphine.paolantoni@enscm.fr (D.P.); sonia.cantel@univ-montp2.fr (S.C.); pascal.dumy@enscm.fr (P.D.)

**Keywords:** dynamic combinatorial chemistry, dynamic covalent chemistry, DNA-templated self-assembly, supramolecular interactions

## Abstract

DNA-templated self-assembly is an emerging strategy for generating functional supramolecular systems, which requires the identification of potent multi-point binding ligands. In this line, we recently showed that bis-functionalized guanidinium compounds can interact with ssDNA and generate a supramolecular complex through the recognition of the phosphodiester backbone of DNA. In order to probe the importance of secondary interactions and to identify side groups that stabilize these DNA-templated self-assemblies, we report herein the implementation of a dynamic combinatorial approach. We used an* in situ* fragment assembly process based on reductive amination and tested various side groups, including amino acids. The results reveal that aromatic and cationic side groups participate in secondary supramolecular interactions that stabilize the complexes formed with ssDNA.

## 1. Introduction

The design of ligands of biomolecules able to establish multiple non-covalent interactions with their targets is of great interest, since it should enable a better and more selective interaction [1,2]. In the field of oligonucleotide recognition, several bifunctional systems, such as bis-intercalators [3,4,5,6,7], small-molecule–intercalators conjugates [8,9], double-headed nucleoside [10] and oligonucleotide [11,12,13,14,15,16]/PNA [17]/polyamide [18,19] conjugates, have been developed in this regard. Furthermore, the multivalent presentation of RNA ligands has been shown to effectively inhibit a key protein-RNA interaction that is involved in myotonic dystrophy [20,21,22,23]. The existence of side groups participating in secondary interactions has also been described at the base pair level. For instance, a modified cytosine analogue capable of “clamp-like” binding through a combination of Watson–Crick pairing and hydrogen bonding on the Hoogsteen edge of the base pair [24,25,26,27,28] has been shown to confer enhanced potency to antisense oligonucleotides [29]. Similarly, it has recently been shown that the presence of additional side-groups that enhance π-stacking interactions greatly improves the selectivity of a novel nucleobase analogue for its complementary partner [30]. The conjugation of an artificial nucleobase to amino acid side groups has also been used to generate potent and selective binders of trans-activation responsive region (TAR) RNA [31,32]. These examples illustrate the growing need to develop multi-point binding ligands of oligonucleotides.

However, the identification of side-groups that participate in stabilizing a ligand-target complex is still a tedious task, as it involves the successive synthesis, isolation and evaluation of a rather large number of candidate compounds. Dynamic covalent chemistry [33] has recently emerged as an attractive tool to generate complex dynamic combinatorial libraries of multifunctional constituents in a one-pot process starting from functionalized fragments. The characterization of the constitutional reorganization that takes place in the presence of a biomolecular template enables the identification of fragments that stabilize the ligand-target complex [34,35,36,37,38,39,40,41,42,43]. For instance, following their first implementation of dynamic combinatorial chemistry (DCC) to identify ligands of DNA and RNA [44,45], Miller and coworkers recently used this technology to identify peptidic dimers as inhibitors of the (CUG) repeat RNA-MBNL1 protein interaction [46]. The group of Balasubramanian also used DCC to identify peptide-intercalator [47] and polyamide conjugates [48] that selectively bind G-quadruplexes. Other recent examples [49] show that DCC is a promising approach to probe secondary interactions and to identify side-groups that participate in biomolecular recognition [34].

**Scheme 1:**
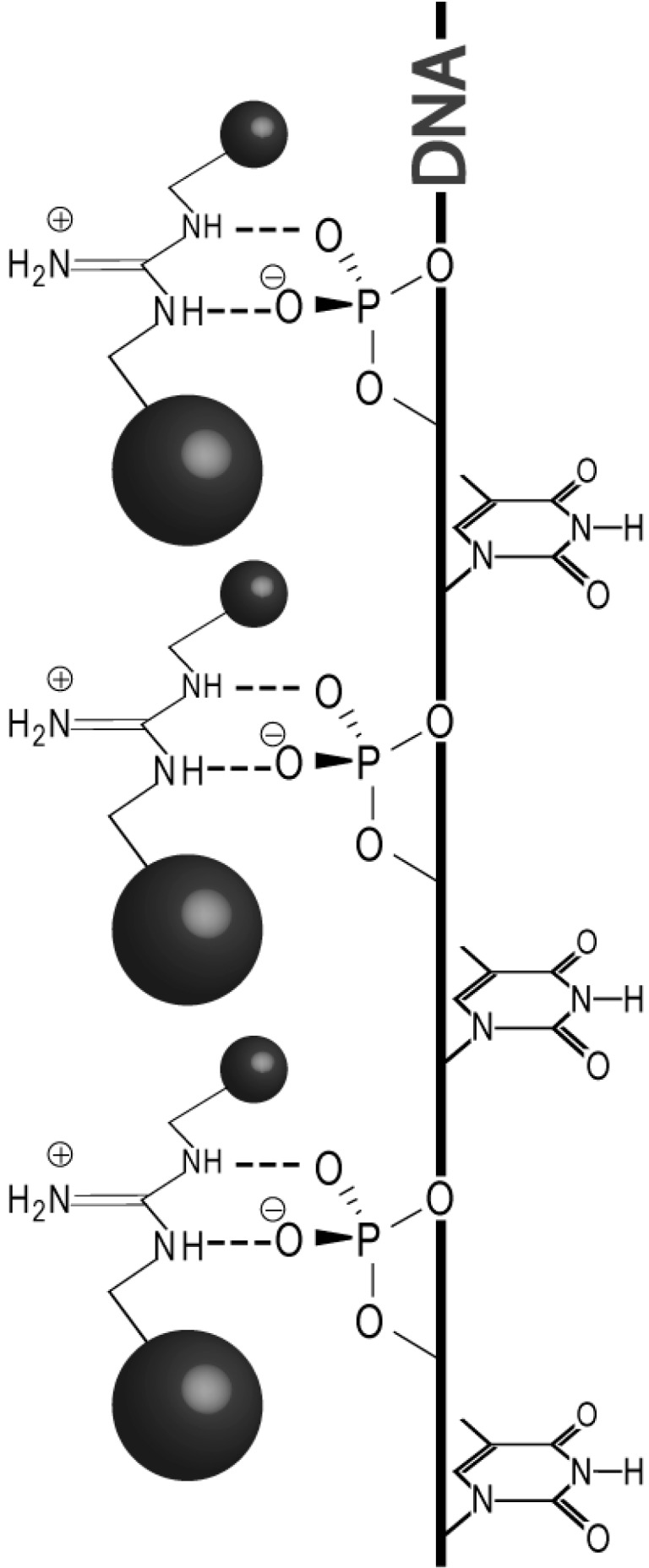
Schematic representation of the single-stranded DNA-templated self-assembly of *bis*-functionalized guanidinium ligands that recognize the phosphodiester backbone of DNA through salt bridges. The presence of side-groups (represented as spheres) may affect the stability of these self-assemblies through self-association or secondary interactions with the ssDNA target.

Besides the recognition of oligonucleotides for therapeutic applications, the development of multi-point binding ligands also features a strong interest from a nanotechnology standpoint. Indeed, oligonucleotides are extensively used to generate functional self-assemblies through either spontaneous DNA assembly and folding processes [50] or templated supramolecular self-assembly of small molecules [50,51,52,53,54,55,56,57,58,59]. The multivalent scaffolding of multiple compounds onto an oligonucleotide array may lead to the emergence of optical [55,60] or recognition [61,62,63,64] properties. In this line, we have recently reported that bis-functionalized guanidinium compounds can interact with ssDNA and generate a supramolecular complex through the recognition of the phosphodiester backbone of DNA [65]. We herein report the implementation of a dynamic combinatorial approach for screening different side-groups and identifying those which stabilize these DNA-templated supramolecular complexes through secondary interactions (Scheme 1).

## 2. Results and Discussion

### 2.1. Design

**Scheme 2:**
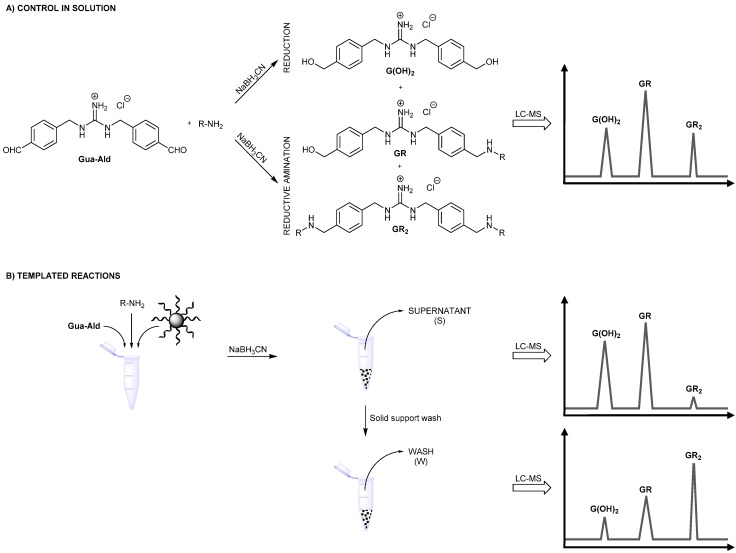
Schematic representation of the* in situ* fragment assembly methodology used for probing the role of side groups. (**A**) Direct reductive amination of Gua-Ald with amines R-NH_2_, in aqueous solution and subsequent analysis by LC-MS; and (**B**) templated reductive amination of Gua-Ald with amines R-NH_2_, in the presence of ssDNA supported on cellulose beads (schematically represented by a regular sphere grafted with ssDNA chains). The subsequent comparative LC-MS analyses of supernatant (S) and wash (W) enable the detection of DNA-binding constituents. Schematic chromatograms illustrate the selective binding of GR_2_ compounds.

We selected a guanidinium compound functionalized with aromatic aldehyde groups as the binding core (Scheme 2A, Gua-Ald). The presence of aldehyde groups should enable the* in situ* generation of imine-based dynamic combinatorial libraries with various amines. The advantages of this method reside essentially in the compatibility between the reductive amination process and nucleic acids, thus enabling* in situ* fragment assembly, and the availability of numerous amines, including amino acids (for reviews on DNA-templated reactions, see for instance [66,67,68]; for examples of DNA-templated polymerization of PNA using reductive amination, see for instance [69]; for examples of DNA-templated polymerization of DNA using reductive amination, see for instance[70]). Although the supramolecular interaction with a related *bis*-functionalized guanidinium ligand devoid of the aldehyde groups seemed weak in solution, MALDI-TOF mass spectrometry analysis clearly showed complex formation in the presence of dT_10_ ssDNA [65]. Furthermore, we previously built dynamic covalent polymers from this building block and showed effective DNA complexation through multivalent interactions [71]. Thus, we reasoned that the presence of two side groups on the guanidinium binding core could considerably impact the stability of the resulting DNA-templated self-assembly. The characterization of this effect through* in situ* fragment assembly should enable the identification of side groups that stabilize these DNA-templated supramolecular complexes (Scheme 2B).

### 2.2. Methodology

Dynamic fragment assembly was performed by direct reductive amination [72] by mixing 1.0 eq. of Gua-Ald with 10.0 eq. of amine (the pH of the stock solution was previously corrected to 7.0) in the presence of 10.0 eq. of sodium cyanoborohydride in MilliQ water (final pH: 5–6). Under these conditions, we observed, by LC-MS, the formation of aminated products, GR and GR_2_, along with the reduced compound, G(OH)_2_ (Scheme 2A). This selectivity between aminated* versus* reduced products (*S*_A/R_) can be quantified as described in Equation (1).

(1) $$S_{\text{A}/\text{R}}=\frac{A_{\text{GR}}+A_{{\text{GR}}_{2}}}{A_{{\text{G}(\text{OH})}_{2}}}$$

where *A*GR, $A_{{\text{GR}}_{2}}$ and $A_{\text{G}{\left(\text{OH}\right)}_{2}}$ represent the HPLC peak area of mono-aminated, *bis*-aminated and reduced product, respectively.

We reasoned that, when the reaction is carried out in the presence of a DNA template, any deviation from this balance between reduced and aminated products should reflect a selective binding of one constituent among the library toward DNA. Thus, we carried out the reductive amination reaction in the presence of dT*_n_* ssDNA supported on cellulose beads and compared the outcome of the reductive amination with the control reductive amination reaction performed in solution, either in the absence of template or with unmodified cellulose. The use of a solid-supported template was motivated by the practical advantage that it confers, that is in terms of the separation of bound constituents* versus* unbound fragments/constituents [45,73,74,75]. Unbound constituents were detected by the direct LC-MS analysis of the supernatant, whereas the treatment of the solid support with 1 M phosphate buffer (pH 6.0) at 60 °C for 10 min and the subsequent LC-MS analysis revealed bound constituents. We determined a retention ratio (*R*_W/S_) that characterizes the selective retention of aminated products on the solid support (Equation (2)).

(2) $$\;R_{\text{W}/\text{S}}=\frac{S_{\text{A}/\text{R}}(\text{wash})}{S_{\text{A}/\text{R}}(\text{supernatant})}$$

### 2.3. Results

Since there was a preliminary indication that π-stacking interactions are stabilizing the ssDNA-templated assembly of guanidinium compounds [65], we first tested aromatic amines, such as benzylamine. In this case, the LC-MS analysis of the supernatant (S) shows the complete disappearance of the bis-aminated product, Gua-Benz_2_ (*S*_A/R_ → 0; Table 1). However, the analysis of the wash solution (W) shows again the presence of this bis-aminated product (*S*_A/R_ = 2.7; Table 1). This result indicates that Gua-Benz_2_ is preferentially retained on the solid support (*R*_W/S_ = 31.4; Table 1). In contrast, an experiment with microcrystalline cellulose alone shows no variation in the S_A/R_ ratios between the supernatant and the wash solutions (*R*_W/S_ = 0.9–1.4; Table 1), thereby indicating that the preferential retention of Gua-Benz_2_ is due to its interaction with dT*_n_*. When carried out in phosphate buffer (pH 6.0) instead of MilliQ water, the retention of the bis-aminated products was much weaker, as indicated by a decrease of the *R*_W/S_ ratio by a factor of eight (*R*_W/S_ = 3.8, Table 1), thus pointing to the key role of salt-bridge interactions in the ssDNA-templated self-assembly of these guanidinium-based compounds (Supplementary Figures S1–S10). MALDI-TOF mass spectrometry analysis of a mixture of Gua-Benz_2_ with dT_10_ (the method to detect non-covalent adducts by MALDI-TOF mass spectrometry was previously developed and optimized using dT10 ssDNA as template, see [65]) shows complex formation, whereas the analysis of a mixture of Gua(OH)_2_ with dT_10_ in the same conditions does not show any complex formation (Supplementary Figures S11–S13). Taken together, these results indicate that the presence of additional benzyl groups greatly enhances the affinity of the corresponding bis-aminated guanidinium compound for dT*_n_* compared to the unfunctionalized guanidinium core alone.

**Table 1:** Reductive amination reaction between Gua-Ald and benzylamine and characteristic values for the selectivity between aminated* versus* reduced products (*S*_A/R_) and for the selective retention of aminated products on the solid support (*R*_W/S_). Errors represent the standard deviation. “*S*_A/R_ (solution)” was determined in the absence of templates.

Conditions	MilliQ H_2_O	Phosphate Buffer	MilliQ H_2_O	Phosphate Buffer
**Template**	**Cellulose**	**Cellulose**	**dT*_n_* on Cellulose**	**dT*_n_* on Cellulose**
*S*_A/R_ (solution)	3.5 ± 0.4	8.1 ± 1.2	3.5 ± 0.4	8.1 ± 1.2
*S*_A/R_ (supernatant)	3.0 ± 0.2	10.4 ± 3.3	0.09 ± 0.01	1.3
*S*_A/R_ (wash)	4.2 ± 0.5	7.6 ± 1.2	2.7 ± 0.5	5.1
*R*_W/S_	1.4 ± 0.1	0.9 ± 0.2	31.4 ± 3.7	3.8

#### 2.3.1. Parallel Screening of Side Groups

**Figure 1:**
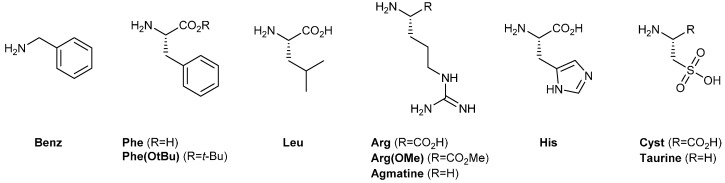
Structures of the amines tested.

A screening of different amines was then undertaken with amino acid derivatives featuring aromatic (Phe), basic (Arg, His), hydrophobic (Leu) and anionic (l-cysteic acid, Cyst) side-chains (Figure 1).

**Figure 2:**
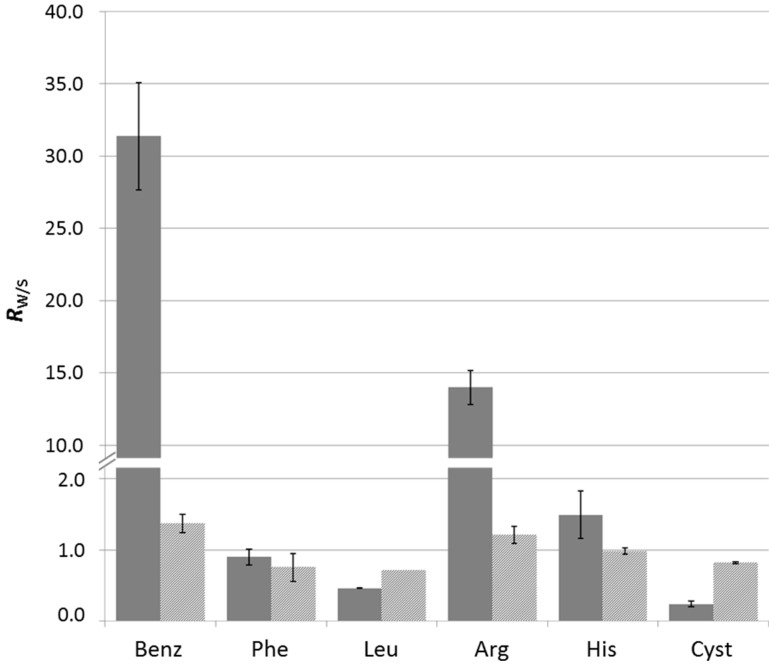
Selective retention of aminated products (*R*_W/S_) with different amino acids in the presence of either cellulose (light grey) or dT*_n_* on cellulose (dark grey). Error bars represent the standard deviation.

The results show that, while microcrystalline cellulose does not generate a selective retention for any amine, Benz and Arg favor the retention of the corresponding GR_2_*bis*-aminated products in the presence of dT*_n_* template (Figure 2 and Supplementary Figures S14–S33). This result indicates that π-stacking and electrostatic interactions are the dominant forces at play in this type of self-assembly. The origin of this effect may be two-fold: (i) the π-stacking interactions may enhance the stability of an arrangement of guanidinium ligands along the phosphodiester backbone of ssDNA through self-association; or (ii) the side group may establish additional supramolecular interactions with the ssDNA template-cationic residues, like Arg, which may, for instance, participate in attractive electrostatic interactions with neighboring phosphodiester residues.

**Figure 3:**
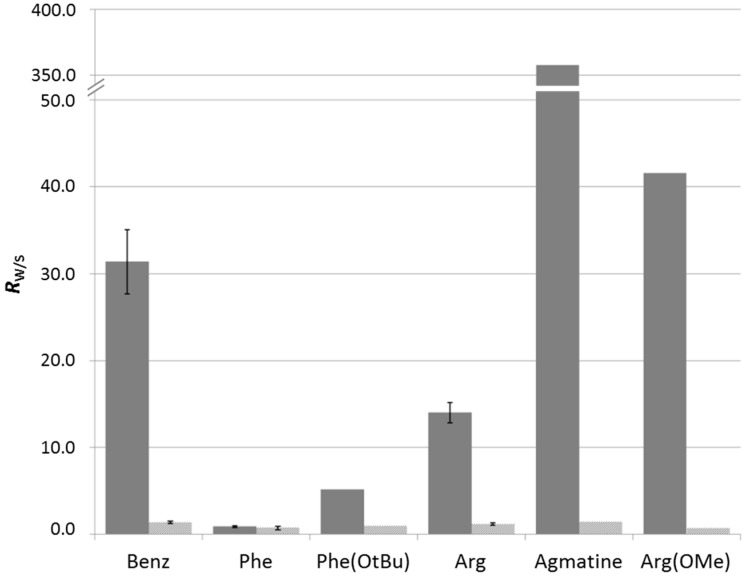
Selective retention of aminated products (*R*_W/S_) with different amines in the presence of either cellulose (light grey) or dT*_n_* on cellulose (dark grey). Error bars represent the standard deviation.

The case of Phe is, at first sight, puzzling, since no selective retention is observed despite the presence of the phenyl moiety that may participate in π-stacking interactions. A possible explanation may be that the negative charge brought by the carboxylic acid moiety prevents association with the ssDNA, due to electrostatic repulsion. Thus, we tested the ester of phenylalanine, Phe(OtBu), and observed a significant retention (Figure 3 and Supplementary Figures S34–S37). Similarly, when comparing Arg with agmatine and Arg(OMe), one can conclude that the presence of the carboxylic acid moiety hinders the interaction with ssDNA (Figure 3 and Supplementary Figures S38–S45).

#### 2.3.2. *In Situ* Screening of Side Groups through Component Selection

In addition to the parallel screening approach based on* in situ* fragment assembly (*vide supra*), the use of reversible covalent chemistry offers the advantage to investigate complex systems in which the selection of optimal components may take place as a result of a recognition process, thereby enabling the one-pot screening of fragments [34,35,38,39,40,41,42,43]. Thus, we studied systems containing two competing amines using the same methodology as described above. The mixtures were analyzed by LC-MS, and the selective formation of aminated products was quantified as described in Equation (3).

(3) $$\;S_{\text{A}1/\text{A}2}=\;\frac{A_{{\text{GR}}^{\text{A}1}}+A_{{{\text{GR}}_{2}}^{\text{A}1}}}{A_{{\text{GR}}^{\text{A}2}}+A_{{{\text{GR}}_{2}}^{\text{A}2}}}$$

where *A*_GR_^A1^ and *A*_GR2_^A1^ represent the HPLC peak area of, respectively, mono-aminated and *bis*-aminated products containing amine A1, while *A*_GR_^A2^ and *A*_GR2_^A2^ represent the HPLC peak area of, respectively, mono-aminated and bis-aminated products that contain amine A2.

In order to quantify the selective retention on the solid support of one aminated product with respect to the other, we determined the selective retention ratio *R*_A1/A2_ (Equation (4)). As defined, this ratio characterizes the selective retention of aminated products containing amine A1* vs.* aminated products containing amine A2.

(4) $$R_{\text{A}1/\text{A}2}=\frac{S_{\text{A}1/\text{A}2}(\text{wash})}{S_{\text{A}1/\text{A}2}(\text{supernatant})}$$

**Figure 4:**
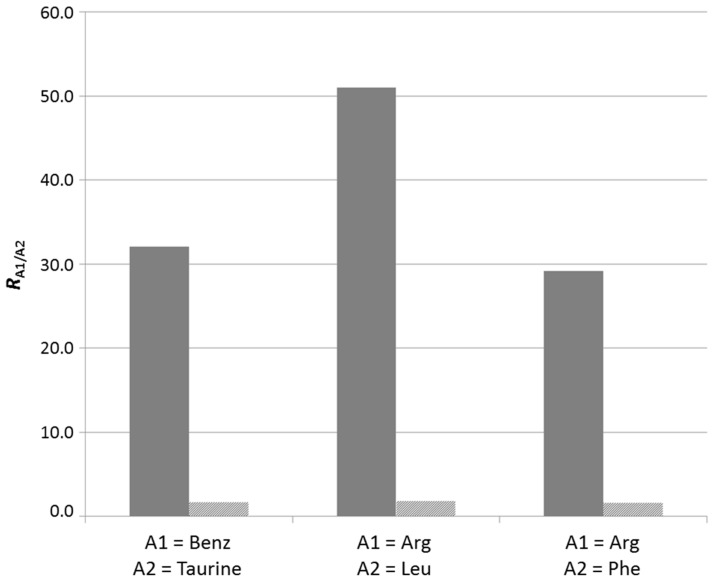
Selective retention of aminated products (*R*_A1/A2_) in competition experiments with two different amines, A1 and A2, in the presence of either cellulose (light grey) or dT*_n_* on cellulose (dark grey).

The results show that benzylamine is selected over taurine during the reductive amination process when the reaction was conducted in the presence of dT*_n_* on cellulose (Figure 4). In contrast, no selection was observed when the reaction was performed with cellulose, thereby indicating that the interaction between ssDNA and the guanidinium compounds is responsible for the observed selection.It is noteworthy to mention that the formation of mixed products containing two different amines was clearly evidenced by LC-MS (Supplementary Figures S46–S55) and shows that the two amines are indeed in competition. Given the results described above, the most likely explanation is that the benzyl side groups provide additional stability, through π-stacking interactions, to the corresponding ssDNA-templated self-assembly and lead to component selection. Similarly, a competition experiment between Arg and Leu shows the preferential selection of Arg, indicating that cationic side groups provide a stabilizing effect, most probably through additional electrostatic interactions with the ssDNA target (Figure 4). Finally, a competition experiment between Arg and Phe point to the superior role of electrostatics compared to π-stacking interactions in this context (Figure 4).

## 3. Experimental Section

### 3.1. General Procedures and Materials

#### 3.1.1. Materials

All reagents and solvents were purchased from commercial sources (Sigma-Aldrich, St. Louis, MO, USA; Alpha Aesar, Ward Hill, MA, USA; or Fisher Scientific, Fair Lawn, NJ, USA) and used as received. Oligo(dT)-cellulose was purchased from Sigma-Aldrich. Oligonucleotides dT_10_ were purchased from Eurogentec (Liege, Belgium) as RP cartridge purification in dried format.

#### 3.1.2. HPLC and LC-MS

Semi-preparative RP-HPLC analyses were performed on Waters instruments (Milford, MA, USA), a 515 HPLC pump connected to Waters 2487 dual λ absorbance detector, with a Macherey-Nagel VP Nucleodur C18 column (HTech 7 µm, 250 mm × 21 mm). LC-MS analysis were performed either on Waters instruments, a 2695 HPLC separation module equipped with a Nucleosil C18 column (Macherey-Nagel, 300 Å, 5 µm, 125 mm × 3 mm), connected to a Waters 996 photodiode array detector and a Waters micromass ZQ mass spectrometer (LC-MS 1) or on a Shimadzu LCMS-2020 equipped with a Kinetex C18 column (Phenomenex, 100 Å, 2.6 µm, 75 mm × 3 mm) (LC-MS 2). For LC-MS 1, Eluent A is water/TFA 99.9%/0.1% and Eluent B is acetonitrile/water/TFA 90%/9.9%/0.1%. The flow is 1 mL/min, and the column is heated at 30 °C. The LC method follows a linear gradient of Eluent B in Eluent A, 5% B → 30% B in 40 min (Method I). For LC-MS 2, Eluent A' is water/TFA 99.9%/0.1% and Eluent B' is acetonitrile/TFA 99.9%/0.1%. The flow is 1 mL/min, and the column is heated at 40 °C. The LC method follows a linear gradient of Eluent B' in Eluent A', 5% B' → 30% B' in 45 min (Method II) or 5% B' → 100% B' in 5 min (Method III).

#### 3.1.3. Mass Spectrometry

Mass spectrometry analyses were carried out on instruments located at the Laboratoire de Mesures Physiques, IBMM, Université de Montpellier, on a Waters Micromass QTof mass spectrometer (positive mode). High resolution mass spectrometry analyses were performed on a Waters Micromass QTof mass spectrometer (positive mode). MALDI mass spectrometry analyses were performed on an Ultraflex III TOF/TOF instrument (Bruker Daltonics, Wissembourg, France) equipped with LIFT capability. A pulsed Nd:YAG laser at a wavelength of 355 nm was operated at a frequency of 100 Hz (MS data). According to the dried droplet procedure, 0.5 μL of a solution of the 4-NA matrix in ethanol (0.1 M) was deposited on the MALDI target (AnchorChip™, Bruker Daltonics), then mixed with the sample in equal amounts. Sample spots were dried at room temperature. MS analyses were conducted in positive reflectron ion mode with a pulse ion extraction delay of 30 ns. An acceleration voltage of 25.0 kV was applied for a final acceleration of 21.95 kV. Mass spectra were acquired from at least 150 laser shots, over a mass range from 500 to 5000 *m*/*z*. A deflection at 2000 Da could be applied. The laser fluence was adjusted for each studied sample above the threshold for the generation of molecular ions. Data were acquired with the Flex Control software (Bruker Daltonics) and processed with the Flex Analysis software. External calibration was systematically performed with commercial peptide mixture (Calibration Peptide Standard II) in a linear correction calibration.

### 3.2. General Procedure for the Generation and Analyses of Dynamic Combinatorial Libraries

All libraries were prepared by the dilution of stock solutions of each component in the appropriate solvent. The stock solutions were at least 10-times more concentrated than the final concentration of the component in the library. Gua-Ald stock solution was prepared in DMSO at a concentration of 100 mM. Amine stock solutions were prepared by dilution of amine in MilliQ water, neutralization at pH 7 by the addition of hydrochloric acid or sodium hydroxide and completion by MilliQ water to obtain a 100 mM solution.

#### 3.2.1. Reductive Amination Reactions in Solution

In a microtube, MilliQ water (the volume needed to reach a final volume of 300 µL), a 100 mM stock solution of guanidinium Gua-Ald in DMSO (3 µL, final concentration of 1 mM) and the 100 mM stock solution(s) of the chosen monoamine(s) (30 µL, 10 eq.) were successively added. After mixing with vortexing and centrifugation, the library was heated at 60 °C for 10 min in a dry bath, then allowed to slowly cool back to room temperature. After a slight centrifugation, an aqueous 100 mM sodium cyanoborohydride solution (30 µL, 10 eq.) was added to the library, which was then mixed again with vortexing and abandoned to room temperature for the necessary time for the reductive amination to be completed (no more constituents with aldehyde visible in LC-MS, ~10 days). The library was then directly transferred in an LC-MS vial and its composition determined by LC-MS (Method I on LC-MS 1 or Method II on LC-MS 2).

#### 3.2.2. Reductive Amination Reactions with Solid Support

Oligo(dT)-cellulose (23.0 mg) or micro-crystallized cellulose (23.0 mg) was weighted in a microtube. To the solid were successively added MilliQ water or a 100 mM phosphate buffer at pH 6 (the volume needed to reach a final volume of 300 µL), a 100 mM stock solution of guanidinium Gua-Ald in DMSO (3 µL, final concentration of 1 mM) and the 100 mM solution(s) of the chosen monoamine(s) (30 µL, 10 eq.). After mixing with vortexing and light centrifugation, the library was heated at 60 °C for 10 min in a dry bath, then allowed to slowly cool back to room temperature. After a slight centrifugation, an aqueous 100 mM sodium cyanoborohydride solution (30 µL, 10 eq.) was added to the library, which was then mixed again with vortexing and abandoned to room temperature for the necessary time for the reductive amination to be completed (no more constituents with aldehyde visible in LC-MS in the corresponding control in solution, ~10 days). The library was centrifuged, and the supernatant was taken with a syringe and directly filtrated through a PVDF 0.45-µm syringe-filter in an LC-MS vial. The composition of the supernatant was then determined by LC-MS (Method I on LC-MS 1 or Method II on LC-MS 2). To the solid support was added a phosphate buffer 1 M solution (330 µL). After mixing with vortexing, the mixture was heated at 60 °C for 10 min and then immediately centrifuged. The washing solution was taken with a syringe and directly filtrated through a PVDF 0.45-µm syringe-filter in an LC-MS vial for analysis (Method I on LC-MS 1 or Method II on LC-MS 2).

#### 3.2.3. Reductive Amination Reactions with Two Different Amines in Competition

The same procedure was used with the following amounts of amines (the indicated numbers of equivalents are given with respect to the amount of Gua-Ald): benzylamine 10 eq. (30 µL) + taurine 10.2 eq. (30.6 µL); l-arginine 10 eq. (30 µL) + l-leucine 10 eq. (30 µL); l-arginine 9.5 eq. (28.5 µL) + l-phenylalanine 14 eq. (42 µL).

#### 3.2.4. Isolation of *Bis*-Aminated Product Gua-Benz_2_

Guanidinium Gua-Ald (25.2 mg; 0.08 mmol) was dissolved in methanol (7.6 mL). Benzylamine (17 µL, 0.16 mmol) was added, and the solution was stirred at room temperature for 2 h. Sodium borohydride (6.2 mg, 0.16 mmol) was then added to the solution. After overnight stirring at room temperature, the reaction mixture was quenched by the addition of aqueous 1 M hydrochloric acid (4 mL) and concentrated *in vacuo*. The residue was purified by semi-preparative HPLC (linear gradient 5% B → 35% B in 30 min, flow 20 mL/min) and lyophilized to provide the product, Gua-Benz_2_, as a white powder (35.0 mg, 78% yield). LC-MS (LC-MS 2): *t*_R_ = 2.41 min (Method III); extracted *m*/*z* calcd. for C_31_H_36_N_5_^+^ ([M]^+^), 478.30; found, 478.25. HR-ESI-MS (+): *m*/*z* calcd. for C_31_H_36_N_5_^+^ ([M]^+^), 478.2971; found: 478.2957.

#### 3.2.5. Isolation of *Bis*-Reduced Product Gua(OH)_2_ and *Bis*-Aminated Product Gua-Agmatine_2_

Agmatine sulfate salt (69.1 mg, 0.30 mmol) was dissolved in aqueous 1 M phosphate buffer (2 mL, pH 6.0). To this solution were successively added a solution of guanidinium Gua-Ald (10.5 mg, 0.03 mmol) in 1 M phosphate buffer (1.2 mL, pH 6.0) and sodium cyanoborohydride (21.0 mg, 0.33 mmol). After stirring for 45 h at room temperature, the products were separated by semi-preparative reverse-phase HPLC (linear gradient 5% B → 20% B in 30 min, flow 20 mL/min) and lyophilized to provide Gua(OH)_2_ and Gua-agmatine_2_ as white powders. LC-MS (LC-MS 2): Gua(OH)_2_*t*_R_ = 1.98 min (Method III), extracted *m*/*z* calcd. for C_17_H_22_N_3_O_2_^+^ ([M − H]^+^), 300.17; found, 300.15. HR-ESI-MS (+): *m*/*z* calcd. for C_17_H_22_N_3_O_2_^+^ ([M]^+^), 300.1712; found, 300.1711; Gua-agmatine_2_*t*_R_ = 1.83 min (Method III), extracted *m*/*z* calcd. for C_27_H_46_N_11_^+^ ([M − 2H]^+^), 524.39; found, 524.25. HR-ESI-MS (+): *m*/*z* calcd. for C_27_H_46_N_11_^+^ ([M − 2H]^+^), 524.3938; found, 524.3943.

## 4. Conclusions

We used herein an* in situ* fragment assembly process based on dynamic covalent chemistry for studying the importance of side groups on the ssDNA-templated self-assembly of guanidinium compounds. The results show that aromatic and cationic side groups participate in secondary interactions that stabilize the supramolecular complexes formed with ssDNA. Furthermore, the competition experiments show that fragments can be screened in a one-pot process through the target-induced selection of optimal components from the dynamic library. The implementation of a dynamic combinatorial approach is therefore an effective strategy for identifying side groups that stabilize a biomolecular complex through an* in situ* fragment-based assembly process.

## Supplementary Materials

Supplementary materials can be found at http://www.mdpi.com/1422-0067/16/02/3609/s1.
